# A novel dynamic robotic moving phantom system for patient‐specific quality assurance in real‐time tumor‐tracking radiotherapy

**DOI:** 10.1002/acm2.12876

**Published:** 2020-04-13

**Authors:** Takehiro Shiinoki, Fumitake Fujii, Koya Fujimoto, Yuki Yuasa, Tatsuhiro Sera

**Affiliations:** ^1^ Department of Radiation Oncology Graduate School of Medicine Yamaguchi University Ube Yamaguchi Japan; ^2^ Department of Mechanical Engineering Graduate School of Science and Technology for innovation Yamaguchi University Ube Yamaguchi Japan; ^3^ Department of Radiological Technology Yamaguchi University Hospital Ube Yamaguchi Japan

**Keywords:** 3D printing technology, dynamic robotic phantom, quality assurance, real‐time tumor‐tracking radiotherapy

## Abstract

In this study, we assess a developed novel dynamic moving phantom system that can reproduce patient three‐dimensional (3D) tumor motion and patient anatomy, and perform patient‐specific quality assurance (QA) of respiratory‐gated radiotherapy using SyncTraX. Three patients with lung cancer were enrolled in a study. 3D printing technology was adopted to obtain individualized lung phantoms using CT images. A water‐equivalent phantom (WEP) with the 3D‐printed plate lung phantom was set at the tip of the robotic arm. The log file that recorded the 3D positions of the lung tumor was used as the input to the dynamic robotic moving phantom. The WEP was driven to track 3D respiratory motion. Respiratory‐gated radiotherapy was performed for driving the WEP. The tracking accuracy was calculated as the differences between the actual and measured positions. For the absolute dose and dose distribution, the differences between the planned and measured doses were calculated. The differences between the planned and measured absolute doses were <1.0% at the isocenter and <4.0% for the lung region. The gamma pass ratios of γ_3 mm/3%_ and γ_2 mm/2%_ under the conditions of gating and no‐gating were 99.9 ± 0.1% and 90.1 ± 8.5%, and 97.5 ± 0.9% and 68.6 ± 17.8%, respectively, for all the patients. Furthermore, for all the patients, the mean ± SD of the root mean square values of the positional error were 0.11 ± 0.04 mm, 0.33 ± 0.04 mm, and 0.20 ± 0.04 mm in the LR, AP, and SI directions, respectively. Finally, we showed that patient‐specific QA of respiratory‐gated radiotherapy using SyncTraX can be performed under realistic conditions using the moving phantom.

## 1 INTRODUCTION

Stereotactic body radiation therapy (SBRT) has been used in clinical practice for a variety of tumor types and anatomical locations.^1^ However, when treating tumors, particularly those located in the thoracic or the abdominal regions, tumor motion during respiration results in considerable geometric and dosimetric uncertainties in dose delivery. Conventionally, large internal margins (IMs) are required to fully cover the geometric changes that occur during free breathing; such large IMs may result in toxicity to healthy tissue.

**Fig. 1:**
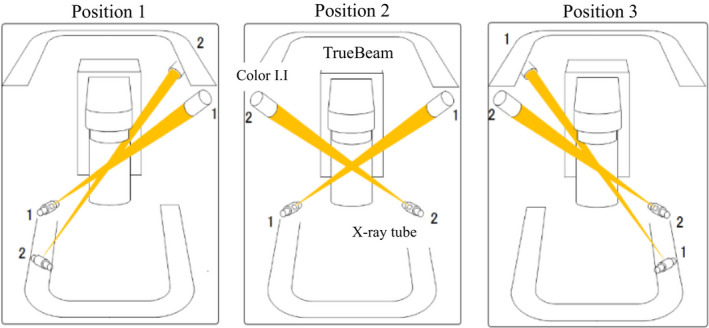
Positions of the x‐ray tubes and color I.I.s can be selected from three options. Therefore, the fiducial markers could be observed using fluoroscopy during radiation treatment with noncoplanar beams. This figure is created based on the graphic user interface of SyncTraX.

At our institution, a novel system that combines TrueBeam (Varian Medical Systems, Palo Alto, CA) and a real‐time tumor‐tracking radiotherapy system called SyncTraX (Shimadzu Co., Kyoto, Japan) was installed to manage tumor motion due to respiration. This system consists of two color image intensifiers (I.I.s) and X‐ray tubes. The color fluoroscopic images are acquired simultaneously from two directions. There are three options for selecting the positions of the X‐ray tubes and I.I.s^2^, ^3^; these positions are indicated in Fig. 1. Thus, fiducial markers implanted near a tumor can be observed using fluoroscopy during radiation treatment with noncoplanar beams.

Color fluoroscopic images acquired along two directions facilitate the automatic extraction of the position of a fiducial marker close to a tumor by a template pattern matching technique in order to calculate the three‐dimensional (3D) coordinates of the markers. When the tracked fiducial marker comes within several millimeters of its 3D planned position (referred to as the gating box), the megavoltage (MV) treatment beam is turned on. This system uses a spatial gating technique that gates the beam by means of the absolute 3D position of the internal fiducial marker instead of using an external surrogate, such as those used in phase or amplitude gating. The details of the SyncTraX system have been reported elsewhere.^2^, ^3^, ^4^ To use current motion management strategies (e.g., breath‐holding, respiratory‐gated radiotherapy, and dynamic tumor tracking radiotherapy technique) in clinical practice,^5^, ^6^, ^7^ a correlation between external markers or sensors and internal tumor motion is required. Many researchers have reported a correlation between external markers and internal tumor motion and have revealed that the maximum variation between the internal tumor motion and the external markers is approximately 10 mm.^8^, ^9^ Although a correlation between external markers and internal tumor positions exists, the external markers cannot adequately indicate the internal tumor positions in some patients. As the SyncTraX system uses the internal fiducial marker for respiratory gating, it is effective in reducing the IMs. This results in a lower dose to normal tissues and, consequently, a lower risk of complications.^10^

In a preliminary study, our group reported that this system can track the motion of a fiducial marker and control radiation delivery with reasonable accuracy.^2^ Flattening filter free (FFF) respiratory‐gated SBRT of a lung tumor was performed using this system in September 2015. We reported that treatment verification in terms of geometric and positional accuracy was achieved in clinical cases using an electronic portal image device and a log file of SyncTraX.^3^ We also reported the imaging dose for real‐time tumor monitoring during respiratory‐gated radiotherapy for the lung using the Monte Carlo technique, but we could not confirm the dose region that may be susceptible to radiation toxicity for the organ at risk.^4^ Moreover, patient‐specific quality assurance (QA) before treatment was not established.

Several researchers have reported patient‐specific dosimetric QA for respiratory‐gated radiotherapy using an external surrogate for commercially moving phantoms.^11^, ^12^ Commercially moving phantoms cannot reproduce the complex 3D respiratory motion. For respiratory‐gated radiotherapy using SyncTraX, the tracking accuracy of the fiducial marker used as an internal surrogate is crucial because the internal surrogate signal is a radiation beam‐on trigger signal.^2^ Furthermore, the size of the gating window and the time delay for respiratory gating are known to cause dosimetric errors.^2^, ^13^ The interfraction and intrafraction changes in the amplitude and speed of the 3D tumor motion affect the efficiency of respiratory‐gated radiotherapy.^14^ To evaluate these uncertainties, a moving phantom is required to realistically reproduce the patient's respiration as well as to mount a dosimetric QA phantom.

It is very important to drive a QA phantom with high accuracy to perform reliable patient‐specific QA. The results of prototype studies on the development of dynamic moving phantoms can be found in the literature. A Stewart platform with six degrees of freedom (DOF) robotic motion phantom using parallel links was proposed^15^ and they subsequently evaluated its positioning accuracy.^16^ A four‐axis moving phantom for patient specific QA, in which four high precision prismatic actuators are used to control the center‐of‐gravity of the phantom, was also proposed recently.^17^ The proposed moving phantom provides sufficiently high accuracy but can be costly. Use of an industrial robotic manipulator for a dosimetric phantom has also been attempted,^18^ and evaluation of its positioning accuracy was performed using the log of the joint actuators of the manipulator. In this case, the pure sinusoidal reference trajectories were evaluated, but the recorded 3D tumor motion of the patient was not tested.

Although some researchers have used a simple water‐equivalent phantom and a 2D array to perform dosimetric verification for respiratory‐gated radiotherapy,^11^, ^19^ such an approach lacks the complex human anatomical information. Recently, 3D printing technology has opened the possibility of customization of a wide variety of applications in the medical field.^20^, ^21^ It is capable of producing individualized lung‐mimicking phantoms and is therefore potentially useful for investigating the accuracy of respiratory‐gated radiotherapy using SyncTraX.

This study was conducted with the objective of assessing a developed novel dynamic moving phantom system that can reproduce patient 3D tumor motion and patient anatomy, and performing patient‐specific QA of respiratory‐gated radiotherapy using SyncTraX.

## 2 METHODS AND MATERIALS

### 2.A Patients and treatment planning

Three patients, who underwent respiratory‐gated SBRT with SyncTraX for a lung tumor, were enrolled in a study by the institutional review board. Three or four fiducial markers with diameters of 1.5 mm (FMR‐201CR; Olympus Co., Ltd, Tokyo, Japan) were implanted close to the tumor in these patients. The clinical characteristics of the patients are summarized in Table 1. All patients were fixed in the supine position on an individualized vacuum pillow (Vac‐Lok system, Civico Medical Solution, Orange City, IA, USA) with their arms raised. For the treatment planning, breath‐hold CT scans were performed with a thickness of 2.0 mm using a 20‐slice CT scanner (SOMATOM Definition AS; Siemens Medical Solutions, Forchheim, Germany) under end‐exhalation, breath‐hold condition. The whole lung was scanned. Breath‐hold duration was about 15 s. The treatment plan was created using a radiation treatment planning system (TPS) (Eclipse version 15.1; Varian Medical Systems). The gross tumor volume was expanded by a margin of 5.0 mm to define the clinical target volume in most instances. Furthermore, a margin of 5.0 mm was added to create a planning target volume or to manually modify it. Six or seven small fields were created. Four of these fields were set to a noncoplanar beam arrangement, whereas the others were set to a coplanar beam arrangement. The beam arrangement is shown in detail in the result section. The prescribed dose was 4800 cGy or 5000 cGy in four or five fractions. The photon beam energy was set to 6 MV‐FFF. In the current version of TrueBeam and SyncTraX, the MV beam of the linac and kilovolt (kV) beam for fluoroscopy must be used simultaneously when the dose rate is greater than 600 MU/min. The MV scatter affects the kV fluoroscopy image quality during simultaneous MV beam and kV fluoroscopy. Therefore, the fiducial marker could not be tracked using the kV fluoroscopy image that is affected by MV scatter. The dose rate was set to 600 MU/min, which is the maximum dose rate that can be used in this combined system. Dose calculation was performed using the anisotropic analytical algorithm.

**Table 1:** Patients characteristics.

Pt. No.	Sex	Age	Tumor location	Prescribed dose (Gy)	Treatment energy	A (mm)				
						LR (mm)	AP (mm)	SI (mm)	3D (mm)	3D TMD (mm)
1	F	81	LLL	10 Gy × 5 fr	6 MV‐FFF	1.7	5.6	17.2	18.2	11.5
2	M	66	RML	10 Gy × 5 fr	6 MV‐FFF	4.5	9.1	11.6	15.4	21.1
3	F	70	RUL	12 Gy × 4 fr	6 MV‐FFF	3.8	5.7	5.4	8.7	10.3

Abbreviations: 3D TMD, 3D tumor‐to‐tracked marker distance;A, peak‐to‐peak amplitude of respiration; AP, Anterior–posterior; F, Female; FFF, Flattening filter free; LLL, Left lower lobe; LR, Left–right; M, Male; RML, Right middle lobe; RUL, Right upper lobe; SI, Superior–inferior.

### 2.B Patients treatment using respiratory‐gated radiotherapy using SyncTraX

Voice coaching was performed throughout patient positioning by a radiological technologist. First, the patient's position was setup at end‐exhalation based on the bone structure using an on‐board imaging (OBI) system at 0° and 90°. Second, the patient was setup at end‐exhalation based on a fiducial marker implanted near the lung tumor using the fluoroscopic images of SyncTraX. Respiratory‐gated radiotherapy was performed using TrueBeam and the SyncTraX system and the patients were treated during free breathing. The gating box was set to 4 mm.

### 2.C Construction of dynamic robotic moving phantom

**Fig. 2:**
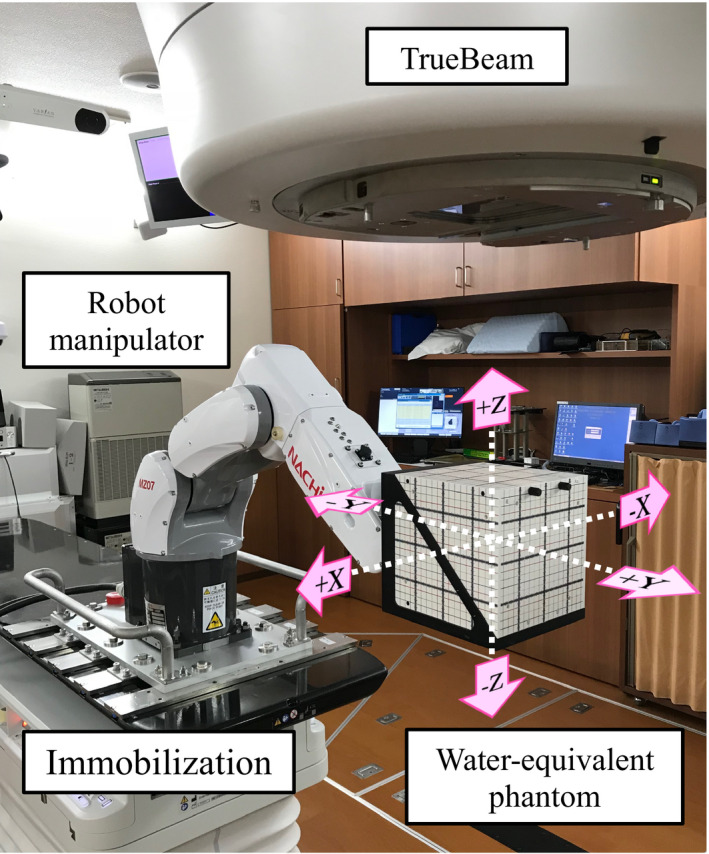
Photograph of the novel dynamic robotic moving phantom and experimental setup. The robot manipulator was immobilized on the treatment couch. The posture of the manipulator was designated. The water‐equivalent phantom (WEP) was set at the tip of the robotic arm. The three axes of motion are along the left‐right (LR, X), superior–inferior (SI, Y), and anterior–posterior (AP, Z) directions.

We focused on a robotic technique to reproduce the 3D respiratory motion. The dynamic robotic moving phantom consisted of a 6‐axis robot manipulator (MZ07‐1; Nachi‐Fujikoshi Co., Ltd., Tokyo, Japan) with a repetitive positioning accuracy of 0.02 mm and a maximum reach 723 mm. Table 2 summarizes the technical specification of the 6‐axis robot manipulator used in this study. *J_i_* (i = 1,2, … ,6) in the table denotes the *i*‐th joint axis. Figure 2 shows a photograph of the novel dynamic robotic moving phantom and experimental setup. The robot manipulator was immobilized on a treatment couch. The posture of the manipulator was designated as shown in Fig. 2. This was decided to avoid beam attenuation by robotic arm when the noncoplanar beam was used for treatment. A water‐equivalent phantom (WEP) weighing 6.1 kg (I'mRT; IBA Dosimetry GmbH, Schwarzenbruck, Germany) was set at the tip of the robotic arm. The allowable payload of the robot manipulator was larger than the load of the WEP. The total weight of the robotic manipulator and the WEP was well within the treatment couch's limits. The three axes of motion were along the left–right (LR, X), superior–inferior (SI, Y), and anterior–posterior (AP, Z) directions.

**Table 2:** Technical specification of robot manipulator. *J_i_* (i = 1,2, … ,6) in the table denotes the *i*‐th joint axis.

Kinematic structure	Revolute joints	
Degrees of freedom	6	
Joint actuation	AC servo	
Maximum reach	723 mm	
Maximum range of motion	J_1_	±2.97 rad
	J_2_	−2.36–1.40 rad
	J_3_	−2.37–4.71 rad
	J_4_	±3.32 rad
	J_5_	±2.09 rad
	J_6_	±6.28 rad
Maximum range of motion	J_1_	7.85 rad/s
	J_2_	6.63 rad/s
	J_3_	9.08 rad/s
	J_4_	9.60 rad/s
	J_5_	9.60 rad/s
	J_6_	17.5 rad/s
Allowable payload	wrist	7 kg
Repetitive positioning accuracy	±0.02 mm	
Allowable environmental temperature	0–45°C	
Total mass	30 kg	

### 2.D Creation of 3D‐printed plate lung phantom

**Fig. 3:**
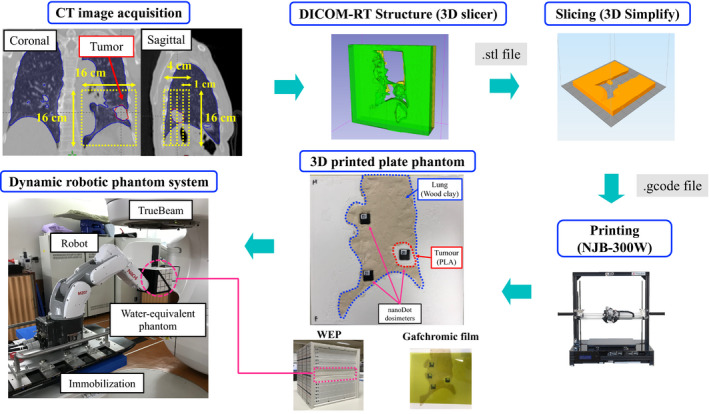
Method for creating the 3D‐printed plate lung phantom. The phantom was designed for planning CT images using the treatment planning system. Four plate phantoms having dimensions of 16 × 16 × 1 cm^3^ were created to include the lung tumor and the fiducial marker used as an internal surrogate. The designed phantom structures were exported as a DICOM‐RT structure file. This file was converted into an STL file using 3D Slicer. The STL file was compiled into G‐code using Simplify 3D software. The patient‐specific phantom was generated on the basis of the G‐code data using a 3D printer with a polylactic acid (PLA) filament. The lung region was filled with wood clay. The two nanoDot OSL dosimeters were set into the lung region and one sheet of Gafchromic film was set in the isocenter plane. Four 3D‐printed plate lung phantoms were inserted into the water‐equivalent phantom, which was set at the tip of the robotic arm.

Figure 3 shows the method followed for creating the 3D‐printed plate lung phantom. The phantom was designed for planning CT images using the TPS. Four plate phantoms with dimensions of 16 × 16 × 1 cm^3^ were created to include the lung tumor and the fiducial marker used as an internal surrogate. This phantom was designed such that the nanoDot optically simulated luminescence (OSL) dosimeter (Landauer, Inc., Glenwood, IL) could be placed inside the lung tumor. The designed phantom structures were exported as a DICOM‐RT structure file. This file was converted into an STL file using 3D Slicer version 4.7. The STL file was compiled into G‐code, which is used to run the commands that modulate the position, velocity, temperature, and extrusion timing of the 3D printer, using slicing software (Simplify3D version 4.0; Simplify3D, Inc., Cincinnati, OH). The printing parameters were determined with reference to a previous study.^20^ The patient‐specific phantom was generated on the basis of the G‐code data using an NJB‐300W personal 3D printer (Ninjabot; LCC, Shizuoka, Japan) with a polylactic acid (PLA) filament, which is a fused deposition modeling‐based 3D printer.^20^ The lung region was filled with wood clay. The two nanoDot OSL dosimeters were set into the lung region and one sheet of Gafchromic film (EBT‐XD; Industrial Specialty Products, Wayne, New Jersey) was set in the isocenter plane. Four 3D‐printed plate lung phantoms were inserted into the WEP, which was set at the tip of the robotic arm.

### 2.E Reproduce 3D respiratory motion using dynamic robotic moving phantom

**Fig. 4:**
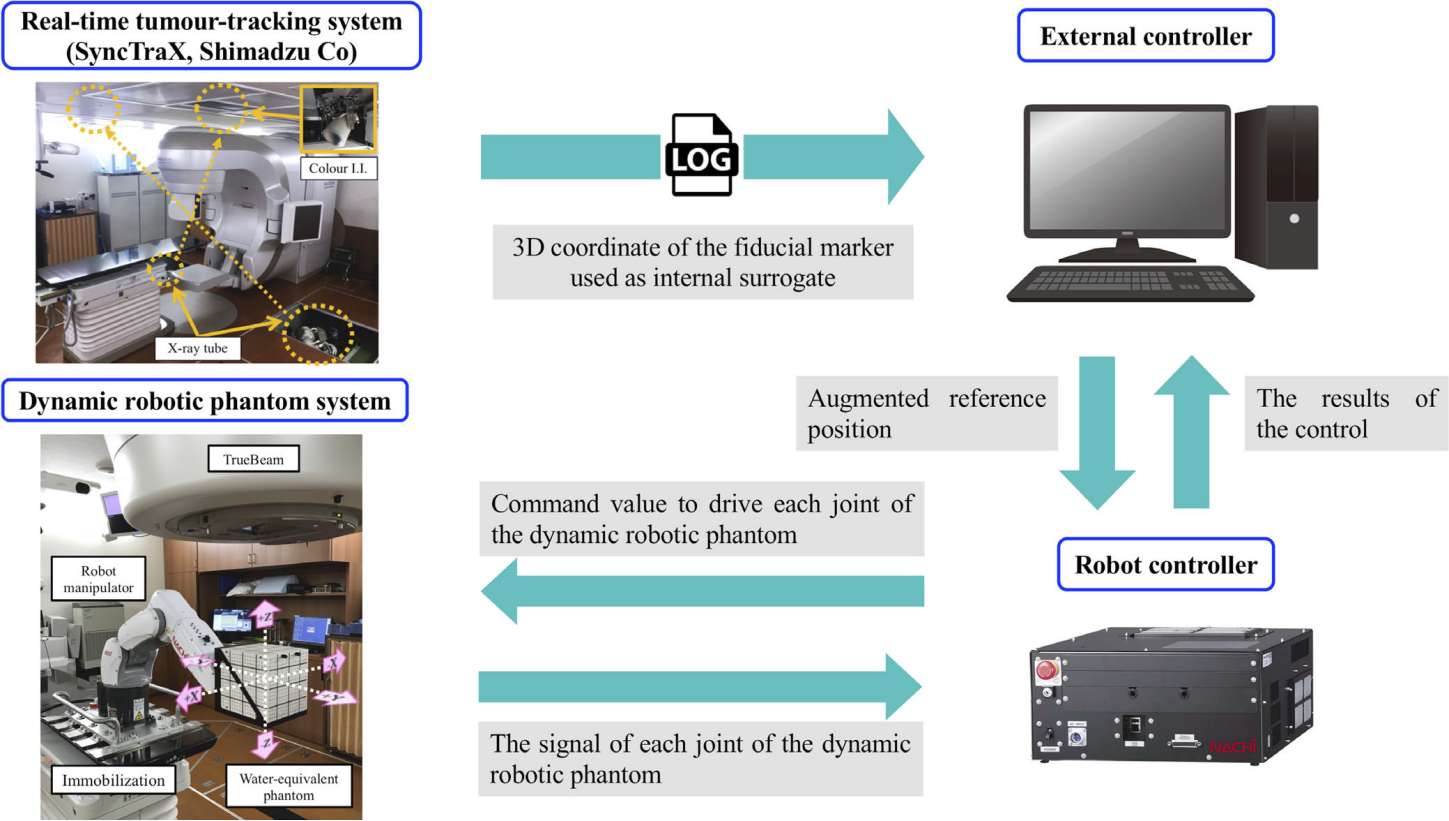
Schematic showing how 3D respiratory motion is reproduced using the developed dynamic robotic moving phantom system.

Figure 4 schematically shows how the 3D respiratory motion is reproduced using the developed dynamic robotic moving phantom system. First, the 3D respiratory motion of the lung tumor was measured using SyncTraX before treatment for each patient. Then, the 3D coordinates of the fiducial marker used as an internal surrogate were recorded at 30 Hz in a log file. Second, the 3D coordinate data were interpolated with the third‐order spline and upsampled to 5 ms to acquire the reference position in order to drive the dynamic robotic moving phantom. For online compensation of the communication delay between the external controller, the robot controller, and the dynamic robotic moving phantom system and the difference between the reference position and the robot tip position, augmented reference position data were acquired using the velocity and acceleration of the interpolated coordinated data.^22^ These data were sent to the robot controller via a TCP/IP connection. Third, the augmented reference position in the treatment room coordinate system was transferred into that in the joint coordinate system of the dynamic robotic moving phantom via inverse kinematics calculation in the robot controller. The command values for driving each joint were sent from the robot controller to the dynamic robotic moving phantom. Then, the dynamic robotic moving phantom reproduced the 3D respiratory motion of the lung tumor. The driving accuracy of the dynamic robotic moving phantom was <0.5 mm.^22^ Finally, driving signals of each joint of the dynamic robotic moving phantom were sent to the robot controller. Then, these signals in the joint coordinate system were transferred into those in the treatment room coordinate system via forward kinematics calculation, and they were recorded in a log file.

### 2.F Reproduction accuracy of patient anatomy for the 3D‐printed plate lung phantom

To evaluate the reproduction accuracy of patient anatomy for the 3D‐printed plate lung phantom, the volume, Hounsfield unit (HU), and mass density of the tumor and the lung of the 3D‐printed plate lung phantom were measured using the TPS and compared with those of the actual patient CT images for all the patients.

### 2.G Patient‐specific dosimetric QA for respiratory‐gated radiotherapy using SyncTraX

For patient‐specific QA, the patient treatment planning described in Section II.A. was copied for the WEP with the 3D‐printed plate lung phantom inserted. Respiratory‐gated radiotherapy with SyncTraX was delivered for a driving dynamic robotic moving phantom that reproduced the 3D respiratory motion. Three nanoDot OSL dosimeters and one sheet of Gafchromic film were inserted in the coronal plane at the isocenter of the WEP. The gating box was set to 4 mm^2^. The tube voltage, current condition, and duration of fluoroscopy were set to 100 kV, 63 mA, and 4 ms, respectively. These conditions enabled us to recognize the fiducial marker in the WEP.

For dosimetric QA, the absolute dose and dose distribution were measured using the nanoDot OSL dosimeters and the Gafchromic film. The measured dose was compared with the planned dose calculated using the TPS. Furthermore, the dose distributions were measured for the driving phantom without respiratory‐gated radiotherapy to evaluate the efficiency of motion management.

For absolute dose measurement, the nanoDot OSL dosimeters used in this study come labeled with their individual sensitivity factors, with accuracies of ±5%. To acquire the calibration curve, the nanoDot OSL dosimeters were irradiated to doses ranging from 50 to 1300 cGy, that is, at 50 cGy intervals up to 300 cGy and at 100 cGy intervals up to 1300 cGy. The photon beam energy was set to 6 MV. Irradiation was performed at a depth of 9 cm with a setup field of 10 × 10 cm^2^ and a source‐axis distance of 100 cm. To ensure adequate photon backscatter, 9 cm of the WEP was set under the dosimeters. The irradiated nanoDot dosimeters were scanned using Microstar II reader (Landauer, Inc., Glenwood, IL) five times. These processes were repeated three times to improve the statistics. The calibration curves were acquired by dividing the range from 0 to 300 cGy and 300 to 1300 cGy by fitting a linear curve.

For planned absolute dose, the nanoDot OSL dosimeters were contoured using TPS. For each contoured nanoDot OSL dosimeter, the mean ± standard deviation (SD) of the planned absolute dose was calculated using TPS.

**Fig. 5:**
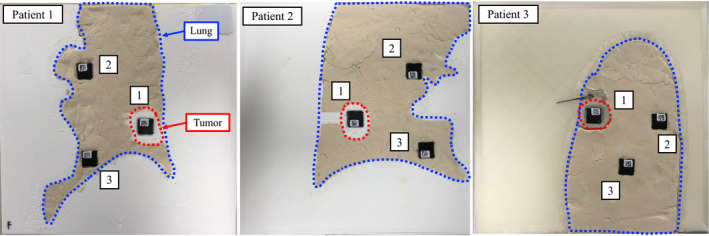
3D‐printed plate lung phantom in the isocenter plane, where the numbers denote the individual nanoDot OSL measurements at the numbered locations.

Figure 5 shows the 3D‐printed lung phantom in the isocenter plane, where the numbers denote individual nanoDot OSL measurements at the numbered locations. The nanoDot OSL dosimeters were located in the high‐dose region with low‐dose gradient while checking the dose distribution using the TPS. The counts acquired from the nanoDot OSL dosimeters irradiated by respiratory‐gated radiotherapy with SyncTraX were converted into absolute doses using the calibration curve. To measure the absolute dose using nanoDot OSL dosimeters, the high efficiency mode was used in a controlled setting because the measurement was considered for clinical application ^23^. The measured absolute dose ( D ) using a nanoDot OSL dosimeter was calculated using the following equation;

(1) $$D=\left(a*\frac{\bar{M}}{S}+b\right)*V$$

where $\bar{M}$ is the average of three readings corrected for readout depletion, V is the daily variation factor calculated from the ratio of the daily standard reading with original standard reading, S is the manufacture‐reported sensitivity of the nanoDot OSL dosimeters, and a and b are the slope and intercept of the appropriate calibration curve.

The absolute dose measurements were performed three times. The mean ± SD of the absolute dose was calculated for each nanoDot OSL dosimeter and compared with the planned absolute dose.

For dose distribution measurement, 18 irradiated films (three × gating or no‐gating × three patients) were scanned in the same orientation (ES‐10000G; Epson Corp., Nagano, Japan) with a resolution of 72 dpi in 48‐bit color scale with a 24‐h postexposure period. All the films were analyzed using commercially available radiation dosimetry software (DD system, version 10.12; R'Tech Inc., Tokyo, Japan). The density of the irradiated films was converted into the absolute dose distribution using a calibration curve; then, the measured and planned dose distributions were compared for an area receiving more than 30% isodose using the gamma index with dose difference/distance‐to‐agreement criteria (γ_D%/dmm_) of 3%/3 mm and 2%/2 mm. The dose distribution was normalized to the maximum dose.

### 2.H Geometric QA for tracking accuracy of SyncTraX system

For geometric QA, the tracking accuracy of SyncTraX was evaluated to compare the measured position with the actual position of the fiducial marker used as an internal surrogate. Respiratory‐gated radiotherapy with SyncTraX was performed for a driven dynamic robotic moving phantom. The log file of the 3D coordinates of the fiducial marker used as an internal surrogate was acquired using SyncTraX. Simultaneously, the results of dynamic robotic moving phantom motion were acquired as a log file. The fiducial marker position recorded using the dynamic robotic moving phantom was defined as the actual position of the fiducial marker and that measured using SyncTraX was defined as the measured position of the fiducial marker. The positional errors between the actual and measured positions of the fiducial marker were calculated in each direction. The root mean square (RMS) values of the positional errors were calculated to evaluate the tracking accuracy of SyncTraX for each treatment field.

## 3 RESULTS

### 3.A Reproducibility of 3D‐printed plate lung phantom

**Fig. 6:**
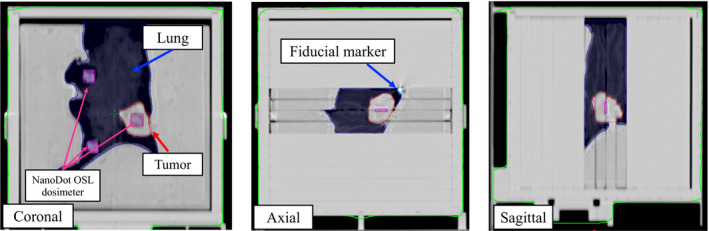
Example of CT images of WEP with 3D‐printed plate lung phantom inserted in the coronal, axial, and sagittal planes for Patient 1.

Figure 6 shows the CT images of the WEP with the 3D‐printed plate lung phantom inserted in the coronal, axial, and sagittal planes for Patient 1. Table 3 summarizes the volume, HU, and mass density of the 3D‐printed plate lung phantoms for all the patients. The corresponding differences between the 3D printed and actual lung tumor volumes were 0.47, 0.05, and 0.40 cm^3^ for each patient. The volumes of the 3D‐printed tumor were consistent with those of the patient CT images. The mean ± SD. of the HU and mass density for the actual and 3D printed tumors were −131.5 ± 92.8 and −122.0 ± 8.2, and 0.91 ± 0.07 g/cm^3^ and 0.92 ± 0.01 g/cm^3^, respectively. Small differences were observed in the HU and mass density of the 3D‐printed tumor. The mean ± SD. of the HU and mass density for the actual and 3D‐printed lungs were −709.7 ± 35.2 and −698.6 ± 33.2, and 0.27 ± 0.04 g/cm^3^ and 0.24 ± 0.04 g/cm^3^, respectively. The HU and mass density of the 3D‐printed lung were nearly consistent with those of the patient CT images. The 3D printing technology could reproduce the patient anatomy with high accuracy.

**Table 3:** Volume, HU, and mass density for 3D‐printed plate lung phantoms of all patients.

Pt.No.	Tumor volume (cm^3^)		Tumor HU		Tumor mass density (g/cm^3^)		Lung HU		Lung mass density (g/cm^3^)	
	Actual	3D printed	Actual	3D printed	Actual	3D printed	Actual	3D printed	Actual	3D printed
1	9.65	9.18	−35.3 ± 183.5	−114.4 ± 138.7	0.98 ± 0.12	0.93 ± 0.16	−748.0 ± 135.4	−736.5 ± 53.0	0.23 ± 0.14	0.20 ± 0.06
2	6.30	6.35	−220.4 ± 257.4	−120.8 ± 166.6	0.84 ± 0.26	0.92 ± 0.20	−678.6 ± 186.7	−675.3 ± 40.1	0.30 ± 0.19	0.27 ± 0.05
3	5.81	5.41	−138.9 ± 220.9	−130.7 ± 145.8	0.92 ± 0.28	0.91 ± 0.17	−702.6 ± 159.7	−683.8 ± 51.8	0.28 ± 0.16	0.26 ± 0.06

Abbreviations: 3D, Three‐dimensional; HU, Hounsfield unit.

### 3.B Patient‐specific dosimetric QA for respiratory‐gated radiotherapy using SyncTraX

Table 4 summarizes the measured and planned absolute doses for each nanoDot OSL dosimeter. The location numbers correspond to those in Figure 5. For all the patients, the differences between the planned and measured absolute doses were <1.0% for the nanoDot OSL dosimeter set into the 3D‐printed tumor. For the other nanoDot OSL dosimeters in the 3D‐printed lung, the differences between the planned and measured absolute doses were <4.0%. For the organ at risk, such as the lung, the measured absolute doses were in good agreement with the calculated ones.

**Table 4:** Measured and planned absolute dose for each nanoDot OSL dosimeter.

Pt. No.	Location	Measured dose (cGy)	Planned dose (cGy)	Difference (%)
1	1	910.6 ± 10.4	917.8 ± 5.3	−0.8
	2	141.5 ± 0.5	135.9 ± 4.7	3.9
	3	161.8 ± 3.1	161.1 ± 11.2	0.4
2	1	824.2 ± 11.2	829.1 ± 3.4	−0.6
	2	132.3 ± 2.6	132.5 ± 3.6	−0.2
	3	135.3 ± 2.6	138.3 ± 4.7	−2.2
3	1	1012.1 ± 7.9	1008.8 ± 3.8	0.3
	2	157.0 ± 4.2	153.6 ± 22.8	2.1
	3	186.6 ± 4.2	191.6 ± 37.0	−2.7

**Fig. 7:**
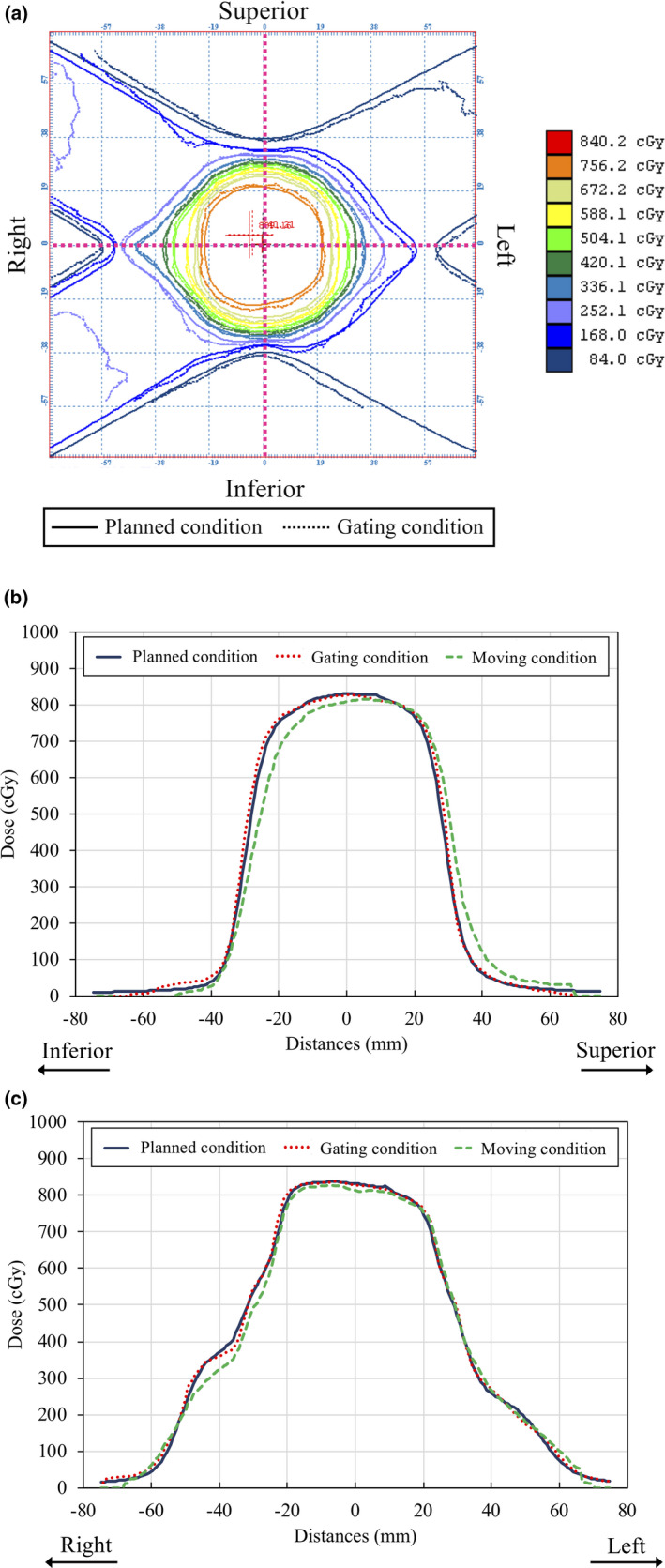
Representative results of patient‐specific QA in the coronal plane. (a) The isodose distribution under the planned condition (solid lines) was compared with that under the gating condition (dash lines). The dose profiles under the planned, moving, and gating condition (solid, dashed, and dotted lines, respectively) are shown along the (b) SI and (c) LR directions.

Figure 7 shows an example of the dose distribution in the coronal plane and the mean dose profiles for three measurements for Patient 2. Although the isodose distribution under the respiratory gating was consistent with the planned isodose distribution, a blurring effect was observed under the no‐respiratory gating in the SI and LR directions. Furthermore, although the respiratory motion was small in the LR direction, the high‐dose gradient region was affected by the blurring effect.

Table 5 compares the planned and measured absolute dose distributions for all the patients. The gamma pass ratios of γ_3 mm/3%_ and γ_2 mm/2%_ under the conditions of gating and no‐gating were 99.9 ± 0.1% and 90.1 ± 8.5%, and 97.5 ± 0.9% and 68.6 ± 17.8%, respectively, for all the patients. The measured dose distributions with respiratory gating were consistent with the planned dose distribution. However, the dosimetric improvement of respiratory gating was small for Patient 3, who had small respiratory motion.

**Table 5:** Comparison between planned and measured absolute dose distributions for all patients.

Pt. No.	Gating (+)		Gating (−)	
	γ_3%/3 mm_	γ_2%/2 mm_	γ_3%/3 mm_	γ_2%/2 mm_
1	99.9 ± 0.1	96.5 ± 1.8	87.4 ± 7.0	61.5 ± 8.9
2	99.9 ± 0.1	98.3 ± 0.3	83.3 ± 4.4	55.4 ± 2.2
3	100.0 ± 0.0	97.6 ± 2.5	99.7 ± 0.2	88.8 ± 8.9

### 3.C Geometric QA for tracking accuracy of SyncTraX system

Table 6 summarizes the RMS values of the positional error in each direction for each treatment field for all the patients. Furthermore, for all the patients, the mean ± SD. of the RMS values of the positional error were 0.11 ± 0.04 mm, 0.33 ± 0.04 mm, and 0.20 ± 0.04 mm in the LR, AP, and SI directions, respectively. The RMS values in the AP direction were larger than those in the other directions because of gravity on the WEP. However, regardless of the gantry, couch angle, and SyncTraX position, the tracking accuracy of SyncTraX was <0.60 mm in all the directions and the 3D direction. The SyncTraX system could track the fiducial marker with high accuracy.

**Table 6:** Root mean square of the positional error in each direction for each treatment field for all patients.

Pt. No.	Field No.	Gantry	Couch	RMS			
				LR (mm)	AP (mm)	SI (mm)	3D (mm)
1	1	330	90	0.08	0.39	0.28	0.49
	2	200	90	0.09	0.27	0.35	0.45
	3	80	25	0.09	0.25	0.22	0.34
	4	70	335	0.19	0.20	0.32	0.42
	5	320	0	0.14	0.22	0.26	0.37
	6	210	0	0.17	0.52	0.12	0.56
2	1	330	90	0.22	0.36	0.21	0.47
	2	200	90	0.17	0.29	0.29	0.44
	3	80	25	0.18	0.30	0.16	0.38
	4	70	335	0.11	0.56	0.15	0.59
	5	320	0	0.19	0.42	0.18	0.50
	6	210	0	0.18	0.39	0.10	0.45
3	1	35	270	0.15	0.37	0.25	0.48
	2	340	270	0.10	0.20	0.21	0.31
	3	165	270	0.09	0.10	0.27	0.31
	4	310	50	0.15	0.26	0.10	0.32
	5	310	325	0.14	0.40	0.08	0.43
	6	150	0	0.11	0.31	0.07	0.34
	7	245	0	0.10	0.41	0.22	0.47
	8	215	0	0.09	0.36	0.08	0.38

Abbreviations: AP, Anterior–posterior; LR, Left–right; RMS, Root mean square; SI, Superior–inferior.

## 4 DISCUSSION

Radiotherapy treatment based on respiratory motion management technology requires an adequate moving phantom for quality assurance.^24^, ^25^ Respiratory‐gated radiotherapy using SyncTraX is currently available in clinical practice. This system uses a spatial gating technique that gates the beam by means of the absolute 3D position of an internal fiducial marker. Therefore, dosimetric and geometric uncertainties should be verified under realistic conditions. We developed a dynamic robotic moving phantom system that reproduces patient 3D tumor motion and patient anatomy to realize patient‐specific QA.

Many moving phantoms have been designed for the QA of motion management. For example, QUASAR (Modus Medical Devices, Inc., London, ON, Canada), CIRS (Computerized Imaging References Systems, Inc., Norfolk, VA, USA), and Dynamic Anatomical Respiring Humanoid Phantom (Radiological Support Devices Inc., Norfolk, VA, USA) are available commercially. Several researchers have developed a moving phantom to perform QA for respiratory motion management.^17^, ^26^, ^27^ All these systems provide interesting solutions to accurately test some aspect of the motion management system. However, most phantoms used are only capable of performing 1D or 2D target motion, or the shapes are simplified and the internal anatomical details are absent. The developed dynamic robotic moving phantom can reproduce the 3D respiratory motion and cover lung tumor motion of up to 34 mm, 24 mm, and 16 mm in the SI, AP, and LR directions, respectively, according to the American Association of Medical Physicists Task Group (AAPM‐TG) 76.^23^ HexaMotion (ScandiDos AB, Uppsala, Sweden) is a five‐dimensional moving platform in combination with Delta4 (ScandiDos AB).^29^ In this study, as the SyncTraX system acquired the 3D coordinates of a single fiducial marker used as an internal surrogate, the developed dynamic robotic moving phantom could only support the 3D tumor motion. However, if tumor rotational information might be acquired, the developed dynamic robotic moving phantom system will also be able to support tumor rotation, such as pitch, roll, and yaw, with 6DOFs (Table 2).

Furthermore, our developed system comprises an industrial robot. As industrial robots are mass produced, cost reduction can be expected. Therefore, our developed system will be less expensive than other phantom systems.

Jung et al. ^30^ developed individualized lung phantoms that can closely mimic the lung anatomy of actual patients using 3D printing technology. The individualized lung inserts and QUASAR respiratory motion phantom were combined to verify the accuracy of CyberKnife tumor‐tracking radiotherapy. We also created 3D‐printed plate lung phantoms using patient CT images and 3D printing technology, and combined them with the developed dynamic robotic moving phantom to confirm the absolute dose, dose distribution, and tracking accuracy for respiratory‐gated radiotherapy with SyncTraX. In particular, the absolute dose into the 3D‐printed tumor could be measured using a nanoDot OSL dosimeter. Our results showed that a lung plate phantom with good similarity to a patient can be manufactured using commercially available 3D printing technology (Fig. 6). The mass densities of the phantom were similar to those of the patients, and the 3D‐printed tumor volumes were nearly consistent with those of the patients. According to AAPM‐TG 101,^1^ treatment‐specific and patient‐specific QA should be established using a moving phantom that simulates respiratory motion. Therefore, we developed a moving phantom that can reproduce the complex 3D respiratory motion and patient anatomy. Then, we established patient‐specific QA for respiratory‐gated radiotherapy with SyncTraX.

However, the 3D‐printed plate lung phantom does not represent the entire human body. The vertebral structures are not included; therefore, dosimetric QA might be easier to achieve than that in an actual situation. Furthermore, the 3D‐printed plate lung phantom could not reproduce the lung deformation due to respiration. In this study, the 3D‐printed plate lung phantoms were created at end‐exhalation. In clinical practice, respiratory‐gated radiotherapy with SyncTraX is performed at end‐exhalation. Thus, patient‐specific QA was performed in a near‐clinical situation.

The positional error perpendicular to the beam axis causes a dosimetric difference compared to the planned dose distribution. The positional error causes blurring in the dose profiles in the case of no‐respiratory‐gated radiotherapy under a moving phantom (Fig. 7). On the other hand, respiratory‐gated radiotherapy with SyncTraX reduced the blurring effect and the measured dose profiles were consistent with the planned dose profile. Our results indicated that the gamma pass ratios of γ_3 mm/3%_ and γ_2 mm/2%_ under the gating condition were 99.9 ± 0.1% and 97.5% ± 0.9%, respectively. These results are comparable with the previous relevant results from CyberKnife tumor‐tracking radiotherapy.^30^

Mutaf et al.^31^ reported that irregular respiratory motion included the baseline shift that introduced critical dosimetric consequences for the target coverage for free breathing. Respiratory‐gated radiotherapy using external markers mitigated the dosimetric impact of the irregularity of patient respiratory motion.^32^ Breath‐hold improved the effect of irregular respiratory motion; however, the reproducibility of breath‐hold is very important.^5^

**Fig. 8:**
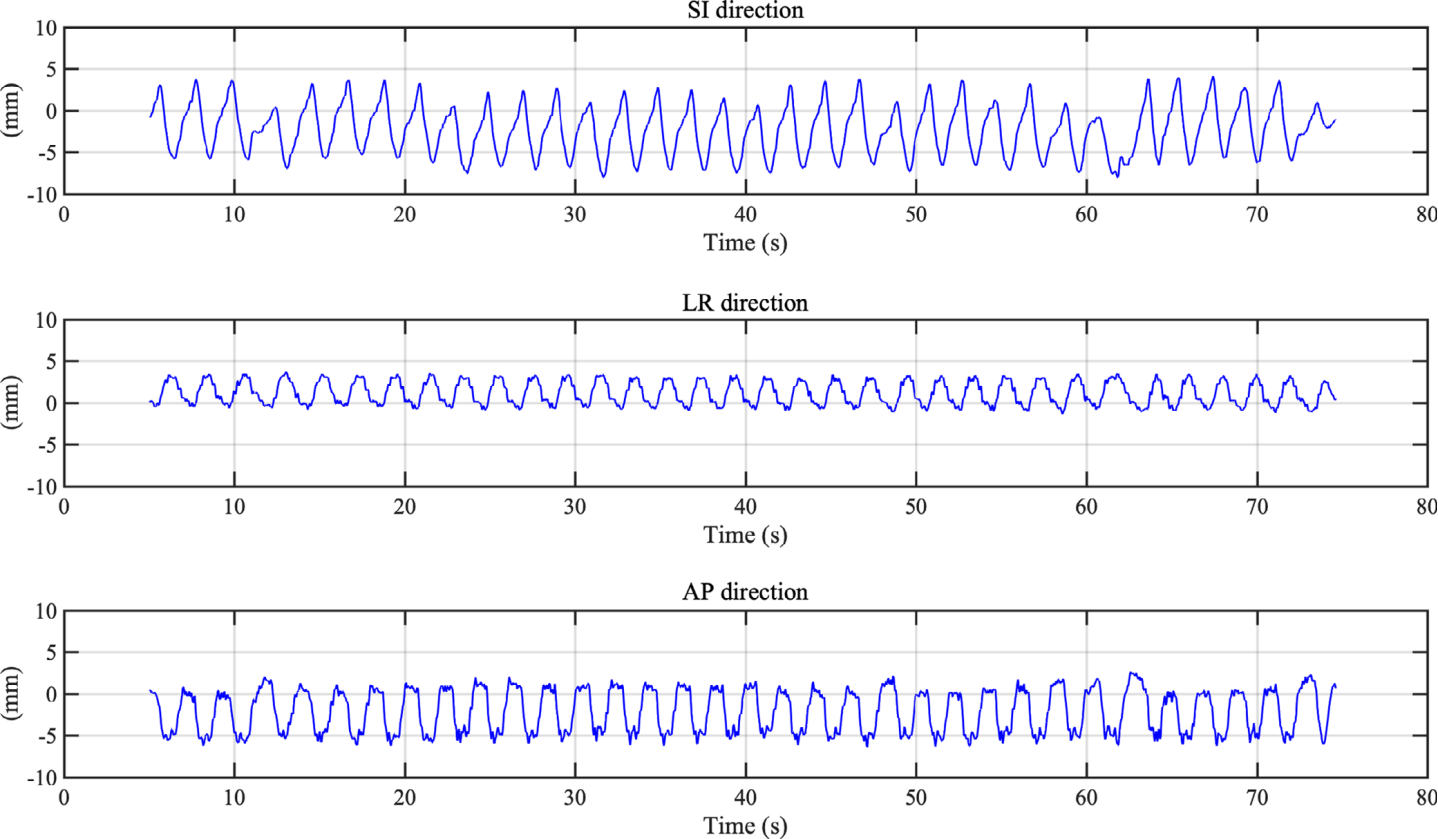
Irregular respiratory motion pattern of Patient 2 in SI, LR and AP directions for patient‐specific QA.

Figure 8 shows the irregular respiratory motion pattern of Patient 2 in SI, LR, and AP directions for patient‐specific QA. Respiratory‐gated radiotherapy with SyncTraX could correct the 3D baseline shift using real‐time tumor monitoring. In this study, respiratory‐gated radiotherapy with SyncTraX reduced the dosimetric impact of 3D irregular respiratory motion using the developed system (Fig. 7).

According to the European Society of Radiotherapy and Oncology guidelines of SBRT, dosimetric accuracy with a median of 3% at the isocenter (2–5%) in a lung phantom inside the treatment field is required.^33^ In this study, the differences between the measured and planned absolute doses into the 3D‐printed tumor inside the treatment field and into the 3D‐printed lung outside the treatment field were <1.0% and <4.0%, respectively, for clinical cases. In our research, nanoDot OSL dosimeters were used to measure the absolute dose in the 3D‐printed plate lung phantom. The nanoDot OSL dosimeters have certain characteristics. Kerns et al.^34^ reported that the angular dependence of the nanoDot OSL should be considered to measure the dose. Lehmann et al.^35^ reported that the angular dependence of the nanoDot OSL dosimeter could be improved by using multiple coplanar beams for clinical measurement, as the overall measurement uncertainty would be reduced. Our measurement results for multiple coplanar and noncoplanar beams in clinical cases were consistent with the results obtained using the TPS.

Ravkilde et al.^36^ evaluated the tracking accuracy of an electromagnetic transponder localization system for dynamic multileaf collimator (DMLC) tracking. They showed that the mean values of the RMS of the positional error for a lung tumor were 0.20, 0.36, 0.28, and 0.56 mm in the LR, AP, SI, and 3D directions, respectively. The developed phantom system enables us to validate not only the tracking accuracy of the SyncTraX system itself but also the absolute dose and dose distribution of respiratory‐gated radiotherapy with SyncTraX. In this study, the tracking accuracy of SyncTraX was <0.60 mm in all the directions, including the 3D direction. These results are similar to the results of Ravkilde et al.^36^ and indicate that the tracking accuracy in clinical cases is extremely high.

Here, the tumor was not tracked using the color fluoroscopic images of SyncTraX. Yamazaki et al.^37^ indicated that the fiducial marker‐tumor misalignment and initial fiducial marker‐tumor distance are related, and when the initial fiducial marker‐tumor distances were within 25 mm, the misalignments were within 2.5 mm. In our study, the 3D distances between the tumor and the fiducial marker used as an internal surrogate were within 22 mm for all patients (Table 1). The geometric relationship between the fiducial markers and the lung tumor could be reproduced using 3D printing technology for all patients. Therefore, as the misalignments between the fiducial marker‐tumor were small, the tracking accuracy could be evaluated for clinical cases.

In the future, we plan to perform respiratory‐gated intensity‐modulated radiotherapy (IMRT) with SyncTraX. The developed phantom system will be useful for performing patient‐specific QA for respiratory‐gated IMRT. Furthermore, it will be useful for the acceptance, commissioning, and QA of novel motion management technologies, such as CyberKnife,^30^ kilovoltage intrafraction monitoring,^36^, ^38^ and DMLC tracking.^39^

However, this developed phantom system has not yet been implemented clinically. Although there are no problems associated with the accuracy of the developed phantom system, a graphic user interface of the phantom control software must be developed to efficiently introduce it to clinical practice.

## 5 CONCLUSIONS

In this study, we developed a novel dynamic robotic moving phantom system that can reproduce patient 3D tumor motion and patient anatomy of the respiratory phase at the time of respiratory gating. Furthermore, we showed that patient‐specific QA of respiratory‐gated radiotherapy using SyncTraX can be performed under realistic conditions using the moving phantom. Overall, the dosimetric and geometric accuracies were found to be sufficiently high in respiratory‐gated radiotherapy with SyncTraX.

## CONFLICT OF INTEREST

The authors have no conflict of interest.
